# Comparing methods for assessing the reliability of health care quality measures

**DOI:** 10.1002/sim.10197

**Published:** 2024-08-15

**Authors:** Kenneth J. Nieser, Alex H. S. Harris

**Affiliations:** 1 Center for Innovation to Implementation, VA Palo Alto Health Care System, Menlo Park, California; 2 Stanford-Surgery Policy Improvement Research and Education Center, Department of Surgery, Stanford University, Stanford, California

**Keywords:** performance measurement, quality measurement, quality of care, reliability

## Abstract

Quality measurement plays an increasing role in U.S. health care. Measures inform quality improvement efforts, public reporting of variations in quality of care across providers and hospitals, and high-stakes financial decisions. To be meaningful in these contexts, measures should be reliable and not heavily impacted by chance variations in sampling or measurement. Several different methods are used in practice by measure developers and endorsers to evaluate reliability; however, there is uncertainty and debate over differences between these methods and their interpretations. We review methods currently used in practice, pointing out differences that can lead to disparate reliability estimates. We compare estimates from 14 different methods in the case of two sets of mental health quality measures within a large health system. We find that estimates can differ substantially and that these discrepancies widen when sample size is reduced.

## 1 | INTRODUCTION

Quality measures are increasingly used by payers to incentivize high-quality care, such as the Center for Medicare and Medicaid Services (CMS) Quality Payment Program and the Hospital Readmission Reduction Programs. Under such programs, hospitals and providers can be financially rewarded or penalized based on their performance on a given set of measures. Quality measures are also increasingly publicly reported, such as on CMS’s Care Compare site, to inform patients of the quality of various providers. Given the high stakes involved and this paradigm’s potential influence on health care, the scientific validity and reliability of these measures are crucial and require a rigorous evaluation process prior to implementation.^1,2^ With these motivations, the Medicare Improvements for Patients and Providers Act of 2008 requires that the U.S. Department of Health and Human Services contract with a Consensus-Based Entity, like the National Quality Forum (NQF) or Battelle, for the independent evaluation of quality measures before use in its programs. These concerns also motivated NQF to establish its Scientific Methods Panel specifically to evaluate measure validity, reliability, and other technical domains of measurement.^1,2^ However, debate and questions remain about which methods should be used to evaluate the validity and reliability of quality measures, as well as how to determine how good is good enough for various uses.^1,3,4^

In this paper, we focus on evaluations of reliability at the accountable entity level—the level at which performance is calculated (eg, providers, hospitals)—as opposed to the data element level—the level at which data used in quality measures are collected (eg, patient-level diagnoses, utilization, patient-reported outcome scales, etc.). At the accountable entity level, reliability quantifies the stability of a measurement if we could hypothetically repeat the measurement again in another sample from the same population of patients and accountable entities in the same time period. In other words, reliability indicates the degree to which observed performance is unaffected by chance factors, like measurement error or sampling error. For example, we might observe a hospital’s risk-adjusted surgical complication rates to be above average, but this could in part be due to random inaccuracies in how data were recorded, abstracted, or analyzed (ie, measurement error) or in which patients happened to visit providers in the evaluated time period (ie, sampling error). Ideally, these chance factors would not greatly impact measurement, especially relative performance between hospitals or providers.

The bulk of methods for quantifying reliability have been developed within the context of educational and psychological testing and measurement at the data element (eg, person) level. In Table 1,we offer a comparison between elements of reliability calculations used at each of the two levels described above—the data element level and the accountable entity level. One important difference between these two settings is that the number and content of items in a test or psychometric scale are the same for every patient; whereas the number of patients each provider cares for and the nature of their health concerns, circumstances, and backgrounds varies markedly. Patients can vary in the degree of attention, time, and resources required to provide necessary care. This distinction has serious ramifications for translating many reliability calculations across levels of inference. For example, calculating Cronbach’s alpha—a common metric of reliability despite its criticisms^5,6^—is based on an estimate of the covariance matrix of items. At the accountable entity level, naively calculating a covariance matrix across patients of different providers would be meaningless. For the same reasons, other factor analytic formulations of reliability commonly employed at the data element level (eg, *ω*) do not seem to directly translate to the accountable entity level.^7^

In response to these challenges, methods have been developed and utilized for quantifying reliability at the accountable entity level;^8–12^ however, disagreement and uncertainty exists among experts regarding the appropriateness of various methods and their interpretations.^1,3,4^ The main contribution of this paper is to provide descriptions and a comparison of common reliability estimation methods to inform the debate regarding this fundamental property of measurement. We review various definitions and methods of estimating accountable entity level reliability. We compare the results of various methods in the case of two empirical examples to demonstrate the sensitivity of reliability estimates to the method chosen. We end with some recommendations.

## 2 | DEFINING RELIABILITY

### 2.1 | Notation

For each provider *i* = 1, …, *m*, we denote the vector of outcomes for the *n_i_* patients of provider *i* as *Y_i_* = (*Y_i_*_1_, …, *Y_in_i__*) and the vector of baseline covariates as *X_i_* = (*X_i_*_1_, …, *X_in__i_*). We note that outcomes could refer to completion of a protocol or process (eg, occurrence of a timely follow-up visit post-hospitalization), as well as patient-level health status (eg, post-surgical infection). For some quality measures (eg, outcome measures), risk-adjustment is needed. Let *θ* be the parameters of the risk-adjustment model used to relate patient baseline covariates to outcomes and $\overset{ˆ}{\theta }$ be estimated parameters.

For each provider *i*, we calculate performance on a quality measure, *T_i_*, which is a function of the outcomes and baseline covariates for each patient. That is, $T_{i}=f\left(Y_{i},X_{i};\overset{ˆ}{\theta }\right)$. For simplicity, in this paper, we focus on the case where risk-adjustment is not needed (eg, process measures) and so below we consider $T_{i}=f\left(Y_{i}\right)$. Often the quality measure is a sample mean, $T_{i}={\overset{‾}{Y}}_{i}$, where ${\overset{‾}{Y}}_{i}=n_{i}^{-1}\sum_{j=1}^{n_{i}}Y_{\mathrm{ij}}$. In the case of binary outcomes, such as whether a patient received follow-up care within 30 days of a hospital discharge or not, the sample mean is a proportion of patients meeting the criteria, which we denote *p_i_*.

### 2.2 | Three formulations of reliability

We review three different statistical formulations of reliability and their corresponding motivations and interpretations.^8^ First, given that reliability aims to capture the stability of a measurement, we can consider a hypothetical scenario in which we have two measurements from the same population, *T_i,_*_1_, *T_i_*_,2_ for each provider *i* = 1, …, *m*. If the two measurements are very similar, we would say the measure is reliable. Otherwise, we would consider the measure to be unreliable. We could quantify this level of agreement with the product-moment correlation,

(1) $$\rho =\mathrm{corr}\left(T_{i,1},T_{i,2}\right).$$

This formulation, which we refer to as a resampling formulation, appeals to the idea that a reliable measure is one that is consistent or stable across multiple measurements. Of course, in practice, we can observe a population of providers and their patients only once. Even if we observe the same providers again, observations would be separated by some time interval over which quality of care might have changed. We discuss this further in the next section.

A second formulation of reliability, which we call the variance ratio formulation, suggests that a reliable measure is one in which a large proportion of the variability in the outcome is explained by variability in provider quality. Under certain conditions, the resampling and variance ratio formulations are mathematically equivalent to each other. To see this, consider the following model for measure *T_i_*:

(2) $$T_{i}=Z_{i}+E_{i,}$$

where *Z_i_* is the underlying quality for provider *i*, and *E_i_* is random error. We assume that quality is uncorrelated across providers, errors are uncorrelated, and that quality is uncorrelated with errors. This model is analogous to classical test theory, where *Z_i_* is the true construct we wish to study (ie, provider quality of care) but our observations are distorted by random errors.^13^ Under this model (2), the reliability definition given in (1) can also be expressed as a variance ratio. Notice that

(3) $$\rho =\frac{\text{cov}\left(T_{i,1},T_{i,2}\right)}{\sqrt{\text{var}\left(T_{i,1}\right)\text{var}\left(T_{i,2}\right)}}\;=\frac{\text{cov}\left(Z_{i}+E_{i,1},Z_{i}+E_{i,2}\right)}{\sqrt{\text{var}\left(T_{i,1}\right)\text{var}\left(T_{i,2}\right)}}\;=\frac{\text{cov}\left(Z_{i},Z_{i}\right)}{\sqrt{\text{var}\left(T_{i,1}\right)\text{var}\left(T_{i,2}\right)}}\;=\frac{\text{var}\left(Z_{i}\right)}{\text{var}\left(T_{i}\right)},$$

where the second equality follows from the model for *T_i_*, the third equality follows from the independence assumptions, and the fourth equality follows because *T_i_,*_1_ and *T_i,_*_2_ are from the same data-generating mechanism. This suggests that we can think of reliability as the ratio of variability in *T_i_* that is due to variability in *Z_i_*. In words, reliability indicates what fraction of the observed variation in measure performance is true variation in quality (ie, signal).

Third, we can also think of reliability as the squared correlation between the true provider quality and the observed performance. This follows from another mathematical equivalence to the previous two definitions:

(4) $${\mathrm{corrr}}^{2}\left(Z_{i},T_{i}\right)={\left(\frac{\mathrm{cov}\left(Z_{i},Z_{i}+E_{i}\right)}{\sqrt{\mathrm{var}\left(Z_{i}\right)}\sqrt{\mathrm{var}\left(T_{i}\right)}}\right)}^{2}\;={\left(\frac{\mathrm{var}\left(Z_{i}\right)}{\sqrt{\mathrm{var}\left(Z_{i}\right)}\sqrt{\mathrm{var}\left(T_{i}\right)}}\right)}^{2}\;=\frac{\mathrm{var}\left(Z_{i}\right)}{\mathrm{var}\left(T_{i}\right)},$$

where the first equality follows from the model for *T_i_* and the second from the independence assumptions. So, we can interpret reliability as the squared correlation between *Z_i_* and *T_i_*. Although these equivalencies hold under the model assumptions given, they might not hold in cases where one or more independence assumptions are violated.

## 3 | ESTIMATING RELIABILITY

We organize approaches for estimating reliability into two groups, based on the first two formulations of reliability discussed in the last section, resampling and variance ratio, respectively. To our knowledge, there have not been methods leveraging the third formulation of reliability—reliability as squared correlation—this would require some method for estimating true provider quality, which is the very object that eludes us. Table 2 provides a summary of the various reliability estimation approaches we discuss next.

### 3.1 | Resampling

The first formulation of reliability, given in Section 2.2, refers to the similarity between two different measurements from the sample population. To operationalize this notion of reliability, we first need to obtain two sets of observations for each provider, which we call *Y_i,_*_1_ and *Y_i,_*_2_. Then, we can calculate measure performance for each set of observations, *T_i,_*_1_ = *f* (*Y_i,_*_1_) and *T_i,_*_2_ = *f* (*Y_i,_*_2_) and evaluate the reliability using a measure of agreement between the two sets. Often, the one-way random effects intraclass correlation coefficient (ICC)^14^ is used, which assumes that the two sets of observations are measuring the same thing and not merely linearly associated as Pearson’s product moment-correlation measures. In other words, reliability is defined as

(5) $$\rho_{\text{resampling}}=\mathrm{ICC}\left(T_{i,1},T_{i,2}\right).$$

The practical challenge with this definition is that we are physically unable to collect a second sample of data for a given population to evaluate reliability. One way of sidestepping this issue is to analyze observations from the same group of providers at a different time (eg, the following calendar year).^15,16^ That is, *T_i,_*_1_ would be calculated based on observations collected in time period 1, and *T_i,_*_2_ would be calculated based on observations collected in time period 2. However, disagreement between observations could be due to systematic changes in provider quality across time periods, rather than chance variations due to measurement or sampling error. For this reason, the quantity calculated in this way—referred to as test-retest reliability—is distinct from the reliability quantity that we aim to capture. With that said, test-retest reliability has been used with the caveat that test-retest reliability is a lower bound on quality measure reliability.^16^

An alternative approach that is commonly used is to split observations for each provider in half and treat each subsample as a sample from the same population.^11,17,18^ This averts concerns about variation in quality over time, because observations from the two subsamples are collected in the same time period. This approach is referred to as split-sample reliability, and we discuss it below.

#### 3.1.1 | Split-sample reliability

To calculate split-sample reliability, we separate each provider’s observations into two parts, *Y_i_* = (*Y_i,_*_1_, *Y_i,_*_2_). Because split-sample reliability is based on half-samples, and reliability depends on sample size, a correction is needed, which can be accomplished with the Spearman-Brown prophecy formula.^19,20^ In addition, we have found that split-sample reliability estimates can greatly depend on the random split of the data, especially in settings with small sample sizes, high within-provider variability, or low between-provider variability.^21^ Repeating the calculation a large number of times *B*, using a different sample split each time, and averaging over these iterations can improve the stability of the split-sample reliability estimator.^21,22^ We refer to this as a permutation split-sample reliability estimate, due to the use of many subsample allocation permutations. We express this estimator as

(6) $${\overset{ˆ}{\rho }}_{\mathrm{PSSR}}=\frac{1}{B}\sum_{b=1}^{B}\frac{2\mathrm{ICC}\left(T_{1}^{\left(b\right)},T_{2}^{\left(b\right)}\right)}{1+\mathrm{ICC}\left(T_{1}^{\left(b\right)},T_{2}^{\left(b\right)}\right)},$$

where $T_{1}^{\left(b\right)},T_{2}^{\left(b\right)}$ are vectors of provider performances calculated using the *b^th^* iteration of splitting the sample into two halves without replacement. The one-way random effects ICC(·, ·) can be calculated using the *psych* package in *R*.^23^

This approach has a couple advantages. First, this method does not rely on any parametric assumptions of the data and so is easily extended to more complicated performance calculations that are not simple means. Second, this method provides a clear reliability estimate for the measure over the given population, unlike many of the variance ratio methods that we discuss next which yield provider-specific reliability estimates. A drawback of this approach is the potentially expensive computation time required to make many splits; though parallel processing provides some relief.

### 3.2 | Variance ratio

Drawing on the second formulation of reliability given in Section 2.2, many variance ratio methods seek to break down the total variability in performance into a component due to true provider quality and a component due to residual variation within providers. To facilitate this, researchers typically assume there is an underlying provider quality random variable *Z_i_* for each provider *i* and hypothesize a model that connects latent provider quality, *Z_i_*, with observed measure performance *T_i_*. With this construction, the variance in observed measure performance can be decomposed using the law of total variance:

(7) $$\text{var}\left(T_{i}\right)=\text{var}\left(E\left[T_{i}|Z_{i}\right]\right)+E\left[\text{var}\left(T_{i}|Z_{i}\right)\right],$$

where the first term captures the variation in provider quality (between-provider variance) and the second term captures within-provider variance. Given this decomposition, reliability can be defined as the proportion of the total variance that is due to the between-provider variance:

(8) $$\rho_{VR}=\frac{\mathrm{var}\left(E\left[T_{i}|Z_{i}\right]\right)}{\mathrm{var}\left(E\left[T_{i}|Z_{i}\right]\right)+E\left[\mathrm{var}\left(T_{i}|Z_{i}\right)\right]}.$$

As the between-provider variation in performance increases relative to the within-provider variation, the reliability increases towards 1. On the other hand, when within-provider variation increases relative to the between-provider variation, reliability tends towards 0. As we discuss below, various methods in this category differ in their modeling assumptions and in how the relevant quantities in (8) are estimated. Most of the current methods in this category used in practice focus on measures that can be expressed as simple averages.

#### 3.2.1 | Analysis of variance

Analysis of variance (ANOVA) is a foundational set of methods that researchers use to decompose variability. For provider *i* and patient *j*, the one-way ANOVA model is

(9) $$Y_{\mathrm{ij}}=Z_{i}+\epsilon_{ij,}$$

where *Z_i_* is the underlying performance for provider *i* with mean *μ* and variance $\sigma_{b,ANOVA}^{2}$, and *ϵ_ij_* is a random error term with mean 0 and variance $\sigma_{w,ANOVA}^{2}$. For measures that can be expressed as simple means, $T_{i}={\overset{‾}{Y}}_{i}$, we can see that $\mathrm{var}\left(E\left[{\overset{‾}{Y}}_{i}|Z_{i}\right]\right)=\sigma_{b,ANOVA}^{2}$ and $E\left[\mathrm{var}\left({\overset{‾}{Y}}_{i}|Z_{i}\right)\right]=\sigma_{w,ANOVA}^{2}/n_{i}$. The ANOVA estimates for $\sigma_{b,ANOVA}^{2}$ and $\sigma_{w,ANOVA}^{2}$ (derivations shown in the Appendix) are

(10) $${\overset{ˆ}{\sigma }}_{b,\mathrm{ANOVA}}^{2}=\frac{\mathrm{MSB}-\mathrm{MSW}}{n_{0}}$$

(11) $${\overset{ˆ}{\sigma }}_{w,\mathrm{ANOVA}}^{2}=\mathrm{MSW}$$

where $n_{0}=(m-1{)}^{-1}\left(N-\sum_{i=1}^{m}n_{i}^{2}/N\right),\mathrm{MSW}=(N-m{)}^{-1}\sum_{i=1}^{m}\sum_{j=1}^{n_{i}}{\left(Y_{\mathrm{ij}}-\bar{Y_{i}}\right)}^{2}$ is the mean square within providers, and MSB $=(m-1{)}^{-1}\sum_{i=1}^{m}n_{i}{\left({\overset{‾}{Y}}_{i}-\bar{\overset{‾}{Y}}\right)}^{2}$ is the mean square between providers.^12^ Plugging these estimators into (8), we obtain an ANOVA-based estimator for reliability:

(12) $${\overset{ˆ}{\rho }}_{\mathrm{ANOVA},i}=\frac{\mathrm{MSB}-\mathrm{MSW}}{\mathrm{MSB}+\left(n_{0}/n_{i}-1\right)\mathrm{MSW}}.$$

Note that due to its dependence on provider-specific sample size *n_i_*, this estimator yields a provider-specific reliability estimate rather than an overall reliability estimate for the population of providers.^24^ This is a common theme for many of the variance ratio approaches that will be described in this subsection. Overall measures of reliability are often obtained by taking the mean or median of all the provider-specific reliability estimates. One benefit of this approach is that point estimates do not rely on any distributional assumptions (eg, Normal distribution).

#### 3.2.2 | Multilevel modeling

Multilevel models, also known as hierarchical or mixed effects models, are modern extensions of the ANOVA models discussed above. Some of the benefits to using multilevel models over simpler ANOVA models include the ease of accounting for missing data and adjustment for covariates. Multilevel models rely on distributional assumptions, and parameters are often estimated with some type of maximum likelihood estimation (eg, restricted maximum likelihood estimation). Below, we review three commonly used multilevel models—hierarchical linear models, hierarchical logistic models, and Beta-Binomial models—as well as corresponding approaches to estimating reliability.

##### Hierarchical linear models

Hierarchical linear models assume that underlying provider quality levels, *Z_i_*, are independently and identically distributed as Normal with mean *μ* and variance $\sigma_{b,\mathrm{HLM}}^{2}$.^25,26^ Given the underlying provider quality level, *Z_i_*, observations are independently distributed according to a Normal distribution with mean *Z_i_* and variance $\sigma_{w,\mathrm{HLM}}^{2}$. Note that the within-provider variance is assumed constant across providers, which could be unreasonable if some providers are more variable in their performance compared to others. A hierarchical linear model can be expressed as:

(13) $$\begin{array}{c} Z_{i}\overset{i.i.d}{∼}N\left(\mu ,\sigma_{b,\mathrm{HLM}}^{2}\right)\\ Y_{i}|Z_{i}\overset{\text{indep}}{∼}N\left(Z_{i},\sigma_{w,\mathrm{HLM}}^{2}\right), \end{array}$$

for each provider *i* = 1, …, *m*. With this model, we have that $\mathrm{var}\left(E\left[{\overset{‾}{Y}}_{i}|Z_{i}\right]\right)=\sigma_{b,\text{HLM}}^{2}$ and $E\left[\mathrm{var}\left({\overset{‾}{Y}}_{i}|Z_{i}\right)\right]=\sigma_{w,\text{HLM}}^{2}/n_{i}$. Using restricted maximum likelihood estimation to estimate ${\overset{ˆ}{\sigma }}_{b,\mathrm{HLM}}^{2}$ and ${\overset{ˆ}{\sigma }}_{w,\mathrm{HLM}}^{2}$, we estimate reliability as:

(14) $${\overset{ˆ}{\rho }}_{\mathrm{HLM},i}=\frac{{\overset{ˆ}{\sigma }}_{b,\mathrm{HLM}}^{2}}{{\overset{ˆ}{\sigma }}_{b,\mathrm{HLM}}^{2}+{\overset{ˆ}{\sigma }}_{w,\mathrm{HLM}}^{2}/n_{i}.}$$

In health care quality measurement, outcomes are usually binary (eg, the follow-up visit occurred or not) and consequently the Normal distribution assumption in the hierarchical linear model would not hold. For this reason, researchers often turn to one of the following two models instead in the case of binary outcomes.

##### Hierarchical logistic models

Like hierarchical linear models, hierarchical logistic models assume that provider quality independently and identically follows a Normal distribution; however, given provider quality, the observed binary outcomes are assumed tobe Bernoulli random variables taking values of either 0 or 1. This can more accurately accommodate binary observations that are common in health care quality measurement. A hierarchical logistic model can be expressed as:

(15) $$Z_{i}\overset{\text{i.i.d}}{∼}N\left(\mu ,\sigma_{b,\text{HLGM}}^{2}\right)\;Y_{i}|Z_{i}\overset{\text{indep}}{∼}\text{Bernoulli}\left({\mathrm{logit}}^{-1}\left(Z_{i}\right)\right),$$

for each provider *i* = 1, …, *m*, where the inverse logistic transformation is applied to provider quality to bound quality between 0 and 1. This transformation introduces additional complications for the estimation. In particular, the between-provider variance parameter estimated with maximum likelihood is on the log-odds scale, rather than the scale of the outcome (ie, probability scale). Hwang et al discuss three different ways of estimating a more interpretable between-provider variance parameter on the probability scale: numerical integration, Monte Carlo integration, and the delta method approximation.^27^ The delta method approximation is the easiest to implement, though could be faulty when sample size is small or between-provider variance is large.^27^ Using the delta method approximation,

(16) $$\mathrm{var}\left(E\left[{\overset{‾}{Y}}_{i}|Z_{i}\right]\right)\approx {\left(\frac{e^{\mu }}{{\left(1+e^{\mu }\right)}^{2}}\right)}^{2}\sigma_{b,\mathrm{HLGM}}^{2}.$$

We can then estimate the between-provider variance using the maximum likelihood estimates $\overset{ˆ}{\mu }$ and ${\overset{ˆ}{\sigma }}_{b,\mathrm{HLGM}}^{2}$. We can interpret $e^{\mu }/\left(1+e^{\mu }\right)$ as an average probability of the outcome in the population. Consequently, instead of using the maximum likelihood estimate, researchers instead might choose to use the overall sample proportion as an estimate for $e^{\mu }/\left(1+e^{\mu }\right)$. We consider both of the options in the case studies in the next section.

One way to avoid the inverse logistic transformation above is to report reliability on the log-odds scale rather than the probability scale.^10^ To better facilitate this, the hierarchical logistic model can be expressed as a latent variable model as follows:

(17) $$Z_{i}\overset{i.i.d}{∼}N\left(\mu ,\sigma_{b,\mathrm{HLGM}}^{2}\right)\;Y_{i}^{*}|Z_{i}\overset{\text{indep}}{∼}Logistic\left(Z_{i},1\right)\;Y_{i}=1\left(Y_{i}^{*}>Z_{i}\right),$$

for each provider *i* = 1, …, *m*, where $Y_{i}^{*}$ is a vector of continuous latent variables, and the observations *Y_i_* take a value of 1 if $Y_{i}^{*}$ exceeds a threshold and is 0 otherwise. Using the fact that the variance of a standardized logistic random variable is equal to π^2^/3, we can estimate reliability as

(18) $${\overset{ˆ}{\rho }}_{\mathrm{HLGM}-log,i}=\frac{{\overset{ˆ}{\sigma }}_{b,\mathrm{HLGM}}^{2}}{{\overset{ˆ}{\sigma }}_{b,\mathrm{HLGM}}^{2}+\frac{\pi^{2}}{3n_{i}}}.$$

While this avoids the additional step of transforming the between-provider variance estimated from the model in (15), reliability estimates on the log-odds scale are not comparable to estimates on the probability scale and tend to be larger.^27^

Returning to reliability estimation on the probability scale, and using the delta method approximation to obtain an estimate of the between-provider variance, another source of variation between methods is the approach to estimating the within-provider variance. One simple and sensible option is to apply the delta approximation and arrive at

(19) $$E\left[\mathrm{var}\left({\overset{‾}{Y}}_{i}|Z_{i}\right)\right]\approx \frac{e^{\mu }}{n_{i}{\left(1+e^{\mu }\right)}^{2.}}$$

Then we can estimate reliability as

(20) $${\overset{ˆ}{\rho }}_{\mathrm{HLGM},i}=\frac{e^{\overset{ˆ}{\mu }}{\overset{ˆ}{\sigma }}_{b,\mathrm{HLGM}}^{2}}{e^{\overset{ˆ}{\mu }}{\overset{ˆ}{\sigma }}_{b,\mathrm{HLGM}}^{2}+{\left(1+e^{\overset{ˆ}{\mu }}\right)}^{2}/n_{i}},$$

However, many researchers calculate provider-specific predictions of variance, such as ${\overset{ˆ}{p}}_{i}\left(1-{\overset{ˆ}{p}}_{i}\right)/n_{i}$ which is based on the variance of a Binomial random variable, where ${\overset{ˆ}{p}}_{i}$ is a sample estimate of performance for provider *i*.^8,9,28–30^ A serious drawback to this is that providers with performance near 0 or 1 will have provider-specific reliability of 1, which is unrealistic.^31^ For this reason, shrinkage estimators, such as the empirical Bayes estimate obtained from a hierarchical logistic regression model, could be used instead.^32–34^ Another more technical but important concern is that the interpretation of the ratio resulting from including provider-specific predictions of variance is not clear. The provider-specific variance predictions are not estimates of the quantity $E\left[\mathrm{var}\left({\overset{‾}{Y}}_{i}|Z_{i}\right)\right]$ but rather predictions of the random variable $\mathrm{var}\left({\overset{‾}{Y}}_{i}|Z_{i}\right)$.^27^ One last concern is that estimating reliability with hierarchical logistic models yields provider-specific reliability estimates, which are often aggregated in an unjustified way, such as taking the mean or median.

##### Beta-binomial model

An alternative to the hierarchical logistic regression model above is the Beta-Binomial model.^8^ Unlike the models above, the Beta-Binomial model assumes a Beta distribution for the underlying provider quality that is bound between 0 and 1. This avoids the need for any transformations of provider quality in the setting of binary outcomes. A Beta-Binomial model can be expressed as:

(21) $$Z_{i}\overset{i.i.d}{∼}Beta(\alpha ,\beta )\;n_{i}{\overset{‾}{Y}}_{i}|Z_{i}\overset{\text{indep}}{∼}Binomial\left(n_{i},Z_{i}\right),$$

for each provider *i* = 1, …, *m*. Under this model, we have that $\mathrm{var}\left(E\left[{\overset{‾}{Y}}_{i}|Z_{i}\right]\right)=\alpha \beta /(\alpha +\beta {)}^{2}(\alpha +\beta +1)$ and $E\left[\mathrm{var}\left({\overset{‾}{Y}}_{i}|Z_{i}\right)\right]=\alpha \beta /n_{i}(\alpha +\beta )(\alpha +\beta +1)$ (derivation in Appendix). This leads to the following Beta-Binomial based reliability estimator,

(22) $${\overset{ˆ}{\rho }}_{\mathrm{BB},i}=\frac{n_{i}}{\overset{ˆ}{\alpha }+\overset{ˆ}{\beta }+n_{i}},$$

where $\overset{ˆ}{\alpha }$ and $\overset{ˆ}{\beta }$ are maximum likelihood estimates of *α* and *β*. This particular method for estimating reliability based on the Beta-Binomial model does not appear to have been recommended in practice to our knowledge.^8^ Instead, as discussed in the case of the hierarchical logistic models, researchers tend to use $\overset{ˆ}{\alpha }\overset{ˆ}{\beta }/(\overset{ˆ}{\alpha }+\overset{ˆ}{\beta }{)}^{2}(\overset{ˆ}{\alpha }+\overset{ˆ}{\beta }+1)$ as an estimate of the between-provider variance and predict the within-provider variance using ${\overset{ˆ}{p}}_{i}\left(1-{\overset{ˆ}{p}}_{i}\right)/n_{i}$. Again, when ${\overset{ˆ}{p}}_{i}$ is 0 or 1, provider-specific reliability estimates will be exactly 1. If enough provider performances are 0 or 1, then the median provider-specific reliability could also be 1, which seems unrealistic.^18^ Some researchers have recently proposed using provider-specific Bayesian estimates using non-informative priors.^35^ They recommend assuming a Jeffrey’s non-informative prior and calculating within-provider variance as ${\overset{ˆ}{p}}^{*}\left(1-{\overset{ˆ}{p}}^{*}\right)/n_{i}$ where ${\overset{ˆ}{p}}^{*}=\left(0.5+\sum_{j=1}^{n_{i}}Y_{\mathrm{ij}}\right)/\left(1+n_{i}\right)$, to address the situations where ${\overset{ˆ}{p}}_{i}$ is 0 or 1. However, the resulting interpretation of the reliability estimate is unclear, given that neither $\overset{ˆ}{p}(1-\overset{ˆ}{p})/n_{i}$ nor ${\overset{ˆ}{p}}^{*}\left(1-{\overset{ˆ}{p}}^{*}\right)/n_{i}$ are an estimate of $E\left[\mathrm{var}\left({\overset{‾}{Y}}_{i}|Z_{i}\right)\right]$. For this reason, we would recommend (22) when using the Beta-Binomial model to estimate reliability.

One practical issue with implementing this approach is that software for estimating the parameters of the Beta-Binomial model is not as readily available and commonplace as that used for hierarchical linear and logistic models. In response, we include sample R code that can be used to estimate parameters of the Beta-Binomial model in the Appendix, and our latest updates can be found here: https://github.com/knieser/quality_measure_reliability. Another concern, again, is that this approach yields provider-specific reliability estimates rather than an overall reliability estimate.

#### 3.2.3 | Resampling-based IUR

One major limitation to the ANOVA and multilevel modeling approaches for obtaining a variance ratio estimate of reliability is that these methods are built for measures that can be expressed as simple averages. In practice, risk-adjusted measures, such as standardized readmission ratios, which are ratios of observed-to-expected outcome rates are not simple averages that are amenable to the modeling approaches described above. To address this gap, a bootstrap resampling-based method has recently been proposed, referred to as resampling-based interunit reliability (resampling-based IUR).^12^ Although this method makes use of resampling, the formulation of reliability ultimately is based on a ratio of between-provider variance to total measure variance, which is why we include itin this section rather than in Section 3.1.

To calculate the resampling-based IUR, first, the overall variance of *T_i_* is estimated using ANOVA formulas by

(23) $${\overset{ˆ}{\sigma }}_{\mathrm{RIUR}}^{2}=\frac{1}{n_{0}(m-1)}\sum_{i=1}^{m}n_{i}{\left(T_{i}-\overset{‾}{T}\right)}^{2},$$

where $n_{0}=(m-1{)}^{-1}\left(N-N^{-1}\sum_{i=1}^{m}n_{i}^{2}\right)$ and $\overset{‾}{T}=N^{-1}\sum_{i=1}^{m}T_{i}$. Then, researchers can generate a large number, *B*, of resamples of data for each provider with replacement and estimate an aggregated within-provider variance as follows:

(24) $${\overset{ˆ}{\sigma }}_{w,\mathrm{RIUR}}^{2}=\frac{\sum_{i=1}^{m}\left(n_{i}-1\right)S_{i}^{*2}}{N-m},$$

where $S_{i}^{*2}=\sum_{b=1}^{B}{\left(T_{i}^{\left(b\right)}-{\overset{‾}{T}}_{i}^{*}\right)}^{2}/(B-1)$ and ${\overset{‾}{T}}_{i}^{*}=\sum_{b=1}^{B}T_{i}^{\left(b\right)}/B$. Lastly, the reliability is estimated as the ratio of between-provider variance—which is the difference between the total variance and the within-provider variance—to the total variance as

(25) $${\overset{ˆ}{\rho }}_{\mathrm{RIUR}}=\frac{{\overset{ˆ}{\sigma }}_{\mathrm{RIUR}}^{2}-{\overset{ˆ}{\sigma }}_{w,RIUR}^{2}}{{\overset{ˆ}{\sigma }}_{\mathrm{RIUR}}^{2}}.$$

Like the Beta-Binomial, there is not a standard, accessible way of calculating reliability in this way. Again, we provide sample R code for calculating this reliability estimate in the Appendix as well as at https://github.com/knieser/quality_measure_reliability. Advantages to this method include its flexibility in what measures it can be applied to, its lack of dependence on data distributional assumptions, and its single overall reliability estimate.

## 4 | APPLICATION TO MENTAL HEALTH QUALITY MEASURES

In this section, we apply the various methods of quantifying reliability to two sets of mental health quality measures related to antidepressant medication management (AMM) and safety planning for Veterans with high risk flags (HRF) for suicide, using national data from the Veterans Health Administration (VA) Corporate Data Warehouse. These measures were chosen primarily for their varying degrees of sample sizes and between-provider variation and for their simplicity—these measures are simple means of binary outcomes and do not require risk-adjustment. For each measure, we compared 14 different ways of estimating reliability, which are summarized in Table 2. Permutation split-sample estimates were based on 200 splits of the data, and resampling IUR estimates were based on 200 bootstrap resamples. For methods yielding provider-specific reliability estimates, we used the median provider-specific reliability. As a sensitivity analysis to this decision, we also repeated analyses using the arithmetic mean. To assess how method comparisons were affected by sample size, we subsampled a fixed percentage of observations from each provider and calculated reliability estimates using these subsamples. To lessen the sensitivity of our results to sampling variation when subsampling, we averaged estimates over 20 iterations of the subsampling for each provider and each fixed percentage. For example, we sampled 60% of the observations, uniformly at random, from each provider 20 separate times. We estimated reliability for each method and iteration and took the average across iterations for each method. All calculations were performed in *R*,^36^ and our code is available at https://github.com/knieser/quality_measure_reliability.

### 4.1 | Antidepressant medication management

Patients who receive a diagnosis of depression and initiate antidepressant medications are recommended to continue medication for a certain period of time to prevent recurrence of a depressive episode. Two related measures from the National Committee for Quality Assurance’s Healthcare Effectiveness Data and Information Set track quality regarding this aspect of psychiatric care. The effective acute phase treatment rate (AMM 1) is the proportion of adults 18 years and older with a diagnosis of major depression and initiate antidepressant medication, who remain on the medication for at least 84 days (12 weeks). The effective continuation phase treatment rate (AMM 2) uses the same denominator of patients but reports the proportion who remain on the medication for at least 180 days (6 months).

**FIGURE 1:**
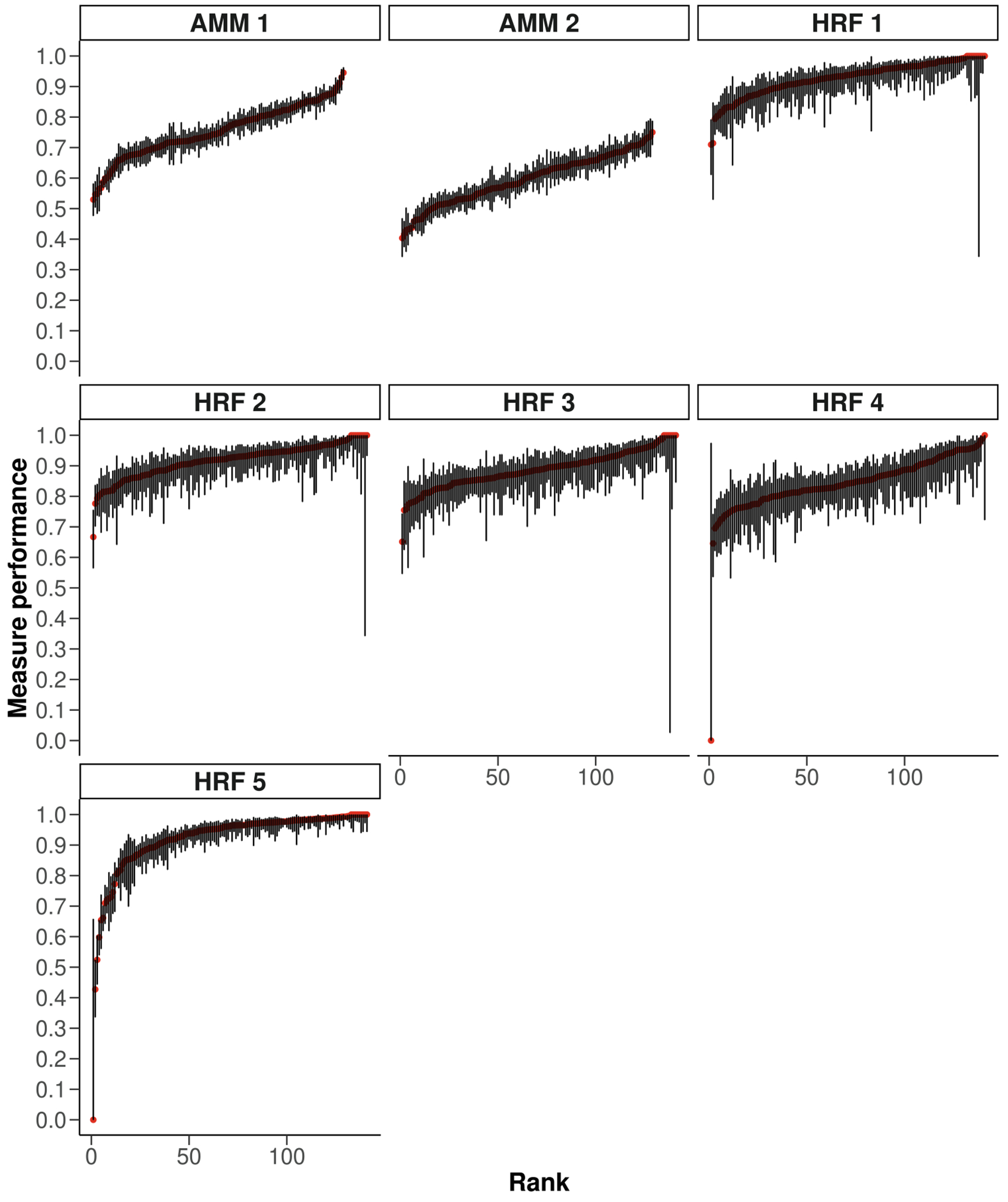
Measure performance by VA medical center shown by red dot with 95% Wilson score confidence intervals. Medical centers are ranked based on estimated performance, which is calculated as a simple sample proportion. Ties are handled by random assignment of rank.

**FIGURE 2:**
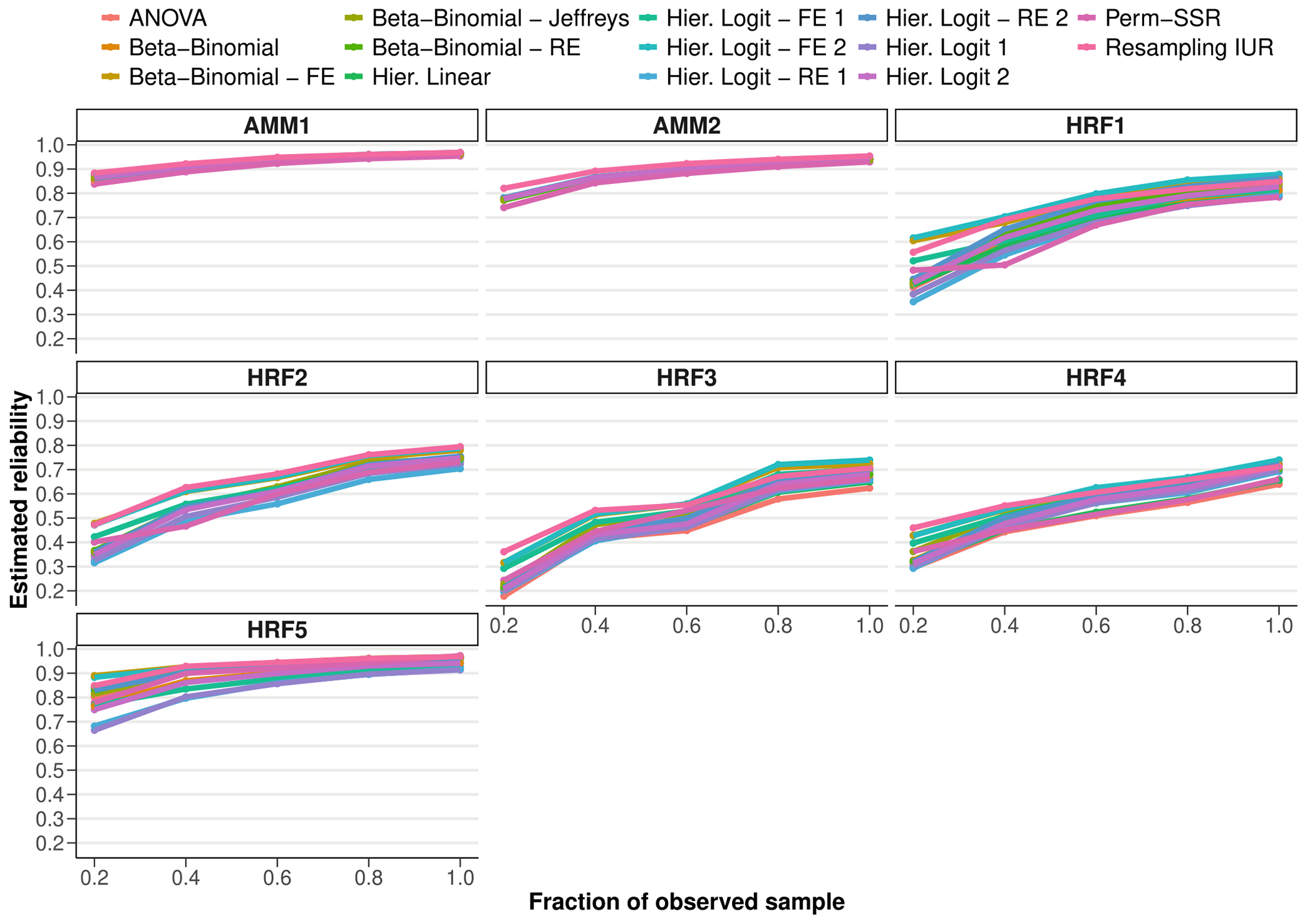
Plots of reliability estimates for each method and for varying sample sizes, corresponding to 20%, 40%, 60%, 80%, and 100% of the observed data for each medical center.

We calculated these two measures for 129 VA medical centers with fiscal year 2017 data. We found large variation across medical centers in the number of patients meeting the measure inclusion criteria, ranging from 159 to 3,279 patients per medical center (more summary details in Table 3). Measure performance varied substantially across medical centers. For AMM 1, performance ranged from 52.5% to 94.3% with a median (IQR) of 74.9% (69.7% to 81.6%). For AMM 2, performance ranged from 40.6% to 74.7% with a median (IQR) of 60.2% (53.2% to 65.4%). Performance estimates and 95% Wilson score intervals are shown in Figure 1. Reliability estimates, shown in Table 4, for both measures were very high (>.93) and consistent across all methods. As sample size decreased, reliability decreased and spread between the estimates from different methods increased (Figure 2). At 20% of the original sample size (a median of 130 patients in the denominator per medical center), reliability estimates ranged from 0.84 to 0.88 for AMM1 and from 0.74 to 0.82 for AMM2. Results using the mean provider-specific reliability were similar and can be found in the Appendix.

### 4.2 | High risk patient flag

Veterans who are perceived by providers to be at high risk for suicide are flagged within the VA’s electronic health record. Suicide Prevention Coordinators at each VA medical center aim to ensure that appropriate and timely care is delivered for Veterans at high risk in an effort to mitigate suicide. VA tracks quality of care delivery in this domain with five related quality measures, which we refer to as HRF1, HRF2, HRF3, HRF4, and HRF5. HRF1 indicates the proportion of Veterans with a new or reactivated flag who have a safety plan documented within 7 days before or after flag initiation, or documented on or before discharge. HRF2 indicates the proportion of Veterans with a new or reactivated flag who received at least four mental health visits within 30 days. HRF3 indicates the proportion of Veterans with a new or reactivated flag who received at least one mental health visit within 31–60 days. HRF4 indicates the proportion of Veterans with a new or reactivated flag who received at least one mental health visit within 61–90 days. HRF5 indicates the proportion of Veterans with a new, reactivated, or continued flag who received a case review between 80 and 100 days for new or reactivated flags or within 7–100 days for continued flags.

We calculated the HRF1 through HRF5 measures for 141 VA medical centers using fiscal year 2019 data. Again, sample sizes of patients meeting denominator criteria varied substantially across medical centers; however, sample sizes were much smaller compared to sample sizes for the AMM measures (summary details in Table 3). Performance varied less across medical centers compared to both AMM measures; performance estimates and associated 95% Wilson score intervals are shown in Figure 1. Given the smaller sample sizes and more moderate variation in performance, reliability estimates were lower for many of the HRF measures, shown in Table 4. Medical centers with only one observation were dropped in the analysis because a minimum of two observations are needed to calculate permutation split-sample reliability without relying on missing data methods. There were more substantial differences in reliability estimates. In particular, for HRF1, reliability estimated with the permutation split-sample method and hierarchical linear model were around 0.79–0.80, while reliability estimated from the hierarchical logistic model using the overall sample proportion in the delta approximation with either fixed or random effects estimates was substantially higher, around 0.86–0.88. As sample size decreased, spread between estimates from different methods increased (Figure 2). For example, HRF1 reliability estimates ranged from 0.78 to 0.88 in the full sample and ranged from 0.35 to 0.62 at 20% of the full sample size (a median of 23 patients in the denominator per medical center). At 20% of the sample size, HRF2 reliability estimates ranged from 0.32 to 0.48, HRF3 estimates ranged from 0.18 to 0.36, HRF4 estimates ranged from 0.29 to 0.46, and HRF5 estimate ranged from 0.66 to 0.89. Results using the mean provider-specific reliability were similar and can be found in the Appendix.

## 5 | DISCUSSION

Quality measures are critical for assessing the level and variation of health care quality across providers and hospitals. Increasingly, quality measures, which are often costly,^37^ are used to incentivize or penalize providers and to make quality gaps among providers and hospitals transparent to patients. For these reasons, it is important that quality measures used to inform these high-stakes decisions are reliable and not an artifact of chance. In this article, we reviewed many methods through which researchers quantify the reliability of health care quality measurements, which we categorize as (1) resampling methods (eg, split-sample reliability) and (2) variance ratio methods (eg, multilevel models). A comparison and discussion of existing methods has not been previously presented in the literature and is necessary to inform measure developers and evaluators on how reliability estimates might depend on the method used. In our two case studies, we found that the various methods could yield substantially different reliability estimates. Differences between methods were largest when sample size was low—a median of 20 to 40 patients per provider. However, rough rules of thumb on minimum sample size are difficult to suggest given that other factors, such as distribution of sample sizes across providers, between-provider variability, and within-provider variability, might also affect estimates.

Several key differences exist between methods of estimating reliability. Major strengths and distinguishing features of the split-sample reliability and the resampling IUR methods are that they (1) do not rely on distributional assumptions about the data; (2) can be calculated for any type of quality measure; and (3) provide an overall, rather than provider-specific, estimate of reliability. Between these two, in our two case studies, split-sample reliability generally provided a lower estimate of reliability, which might be preferable when resources and patient safety are at stake. Among methods based on hierarchical models, we would recommend the versions of the hierarchical logistic model and Beta-Binomial models given in (20) and (22), respectively, which have clear interpretations. While we did not originally set out to innovate on existing methods, the Beta-Binomial model-based reliability estimate we provide in (22) has not been previously described to our knowledge, despite its simplicity and connection to other variance ratio estimates. Some drawbacks of methods based on hierarchical models include their reliance on distributional assumptions about the data which could be incorrect; their limitation to measures that can be expressed as means as opposed to more complicated measures (eg, observed-to-expected ratios); and their resulting provider-specific estimates of reliability which then are typically summarized in some way, such as the median.

Based purely on the number of assumptions required, flexibility, and interpretability, we would recommend the permutation split-sample reliability method over other methods. However, to gain greater confidence in this recommendation, more work will be needed to understand the connections between what low or high reliability estimates from each of these methods conveys in terms of stability of provider ranking and/or classification. One of the key advantages of the permutation split-sample reliability method is the interpretability of the single estimate of reliability it produces. Measure developers and evaluators using methods that yield a distribution of provider-specific reliability estimates generally focus on the mean or median of this distribution; however, other aspects of this distribution might be important to consider, and this is currently unsettled. Using the median as an overall measure of reliability can obscure the fact that reliability is very low for many providers. In addition, provider-specific reliability is often interpreted as an indication of probability of provider misclassification; however, studies have shown otherwise.^4,38^

Related to the last point, higher reliability estimates from *any* of the methods are not necessarily indicative of a greater ability to differentiate between any subset of providers.^34^ Consider performance on HRF1. All methods suggest a reliability of at least 0.78 which exceeds a commonly used threshold of 0.7 for good reliability.^2,33,39^ However, it is clear from Figure 1, that confidence intervals of many medical centers’ performances overlap. Although there is substantial heterogeneity in provider quality across all medical centers, this does not mean that any two medical centers pulled out from this population differ in their quality. This raises a sometimes overlooked point that reliability estimates are dependent on the particular population or subpopulation under examination. Consequently, for example, measures might be highly reliable when reported at a national or overall health system level, but can be much less reliable when reported at a regional or state level.

There are a few limitations to our findings. First, the difference between reliability estimates shown in our case studies were based on just two sets of quality measures from one large health system. Consequently, these findings might vary, to a greater or lesser extent, in other settings. Second, many quality measures, particularly those related to patient outcomes, require risk adjustment to ensure fair comparison of performance across providers and hospitals. Process measures, such as the measures considered above, are generally not risk-adjusted, so we did not discuss risk-adjusted measures in detail in this paper. Adjusting for differences in outcomes across providers and hospitals can reduce between-provider variation, and consequently lower reliability.^40,41^ If risk-adjustment is inadequate, estimates of between-provider variation, and consequently reliability, could be inflated. For this reason, valid risk-adjustment procedures are also necessary to ensure the accuracy of reliability calculations. Various risk-adjustment methods exist^42,43^ and exploration of how various risk-adjustment methods impact the comparison of reliability methods requires further study. For some reliability estimation methods (eg, Beta-Binomial model-based estimates^8,25,35^), little to no guidance is available on the most appropriate ways to incorporate risk-adjustment.

The variation in reliability estimates across different methods poses a significant concern. Preference for one method over might depend on the ultimate purpose of the reliability estimate. In some cases, we are interested in identifying providers that are outliers—those performing substantially worse (or better) than their peers. In this setting, a metric known as the profile inter-unit reliability has been suggested.^44^ Another common application of quality measures is in provider profiling or ranking. This suggests that reliability methods that communicate stability in ranking or classification rather than raw performance scores are important. These questions are fundamentally different from the one that the methods discussed in this paper aim to answer. Future development and selection of methods for calculating reliability of health care quality measures should take context and intention into account.

## Figures and Tables

**TABLE 1:** A comparison of the components of measurement reliability at two different levels of inference.

	Level 1 (Data element level)	Level 2 (Accountable entity level)
Example	PHQ-9^a^	Percentage of patients with a diagnosis of depression who had a documented PHQ-9 score
Construct of interest	Depression severity	Provider’s quality of care
Subject of the measurement	Patient	Provider
Observations	Questionnaire responses	Patients
Aggregate score	Total score	Measure performance

a Patient health questionnaire-9.^45^

**TABLE 2:** Summary of reliability estimators.

Method	Formula	Applicable context
Permutation split-sample	$\frac{1}{B}\sum_{b=1}^{B}\frac{2\mathrm{ICC}\left(T_{1}^{\left(b\right)},T_{2}^{\left(b\right)}\right)}{1+\mathrm{ICC}\left(T_{1}^{\left(b\right)},T_{2}^{\left(b\right)}\right)}$	Any
ANOVA ^a^	$\frac{MSB-MSW}{MSB+\left(n_{0}/n_{i}-1\right)MSW}$	Sample means of outcomes
Hier. Linear ^a^	$\frac{{\overset{ˆ}{\sigma }}_{b,HLM}^{2}}{{\overset{ˆ}{\sigma }}_{b,HLM}^{2}+{\overset{ˆ}{\sigma }}_{w,HLM}^{2}/n_{i}}$	Sample means of continuous, Normal distribution outcomes
Hier. Logit 1^a,b^	$\frac{{\left(\frac{exp\overset{ˆ}{\mu }}{(1+exp\overset{ˆ}{\mu }{)}^{2}}\right)}^{2}{\overset{ˆ}{\sigma }}_{b,HLGM}^{2}}{{\left(\frac{exp\overset{ˆ}{\mu }}{(1+exp\overset{ˆ}{\mu }{)}^{2}}\right)}^{2}{\overset{ˆ}{\sigma }}_{b,HLGM}^{2}+\frac{exp\overset{ˆ}{\mu }}{n_{i}(1+exp\overset{ˆ}{\mu }{)}^{2}}}$	Sample means of binary outcomes
Hier. Logit-FE 1^a,b^	$\frac{{\left(\frac{exp\overset{ˆ}{\mu }}{(1+exp\overset{ˆ}{\mu }{)}^{2}}\right)}^{2}{\overset{ˆ}{\sigma }}_{b,HLGM}^{2}}{{\left(\frac{exp\overset{ˆ}{\mu }}{(1+exp\overset{ˆ}{\mu }{)}^{2}}\right)}^{2}{\overset{ˆ}{\sigma }}_{b,HLGM}^{2}+\frac{{\overset{ˆ}{p}}_{i,FE}\left(1-{\overset{ˆ}{p}}_{i,FE}\right)}{n_{i}}}$	Sample means of binary outcomes
Hier. Logit-RE 1^a,b^	$\frac{{\left(\frac{exp\overset{ˆ}{\mu }}{(1+exp\overset{ˆ}{\mu }{)}^{2}}\right)}^{2}{\overset{ˆ}{\sigma }}_{b,HLGM}^{2}}{{\left(\frac{exp\overset{ˆ}{\mu }}{(1+exp\overset{ˆ}{\mu }{)}^{2}}\right)}^{2}{\overset{ˆ}{\sigma }}_{b,HLGM}^{2}+\frac{{\overset{ˆ}{p}}_{i,RE}\left(1-{\overset{ˆ}{p}}_{i,RE}\right)}{n_{i}}}$	Sample means of binary outcomes
Hier. Logit 2^a,c^	$\frac{{\overset{‾}{p}}^{2}(1-\overset{‾}{p}{)}^{2}{\overset{ˆ}{\sigma }}_{b,HLGM}^{2}}{{\overset{‾}{p}}^{2}(1-\overset{‾}{p}{)}^{2}{\overset{ˆ}{\sigma }}_{b,HLGM}^{2}+\frac{\overset{‾}{p}(1-\overset{‾}{p})}{n_{i}}}$	Sample means of binary outcomes
Hier. Logit-FE 2^a,c^	$\frac{{\overset{‾}{p}}^{2}(1-\overset{‾}{p}{)}^{2}{\overset{ˆ}{\sigma }}_{b,HLGM}^{2}}{{\overset{‾}{p}}^{2}(1-\overset{‾}{p}{)}^{2}{\overset{ˆ}{\sigma }}_{b,HLGM}^{2}+\frac{{\overset{ˆ}{p}}_{i,FE}\left(1-{\overset{ˆ}{p}}_{i,FE}\right)}{n_{i}}}$	Sample means of binary outcomes
Hier. Logit-RE 2^a,c^	$\frac{{\overset{‾}{p}}^{2}(1-\overset{‾}{p}{)}^{2}{\overset{ˆ}{\sigma }}_{b,HLGM}^{2}}{{\overset{‾}{p}}^{2}(1-\overset{‾}{p}{)}^{2}{\overset{ˆ}{\sigma }}_{b,HLGM}^{2}+\frac{{\overset{ˆ}{p}}_{i,RE}\left(1-{\overset{ˆ}{p}}_{i,RE}\right)}{n_{i}}}$	Sample means of binary outcomes
Beta-Binomial^a^	$\frac{n_{i}}{\overset{ˆ}{\alpha }+\overset{ˆ}{\beta }+n_{i}}$	Sample means of binary outcomes
Beta-Binomial-FE^a^	$\frac{\overset{ˆ}{\alpha }\overset{ˆ}{\beta }}{\overset{ˆ}{\alpha }\overset{ˆ}{\beta }+(\overset{ˆ}{\alpha }+\overset{ˆ}{\beta }+1)(\overset{ˆ}{\alpha }+\overset{ˆ}{\beta }{)}^{2}\frac{{\overset{ˆ}{p}}_{i,FE}\left(1-{\overset{ˆ}{p}}_{i,FE}\right)}{n_{i}}}$	Sample means of binary outcomes
Beta-Binomial-RE^a^	$\frac{\overset{ˆ}{\alpha }\overset{ˆ}{\beta }}{\overset{ˆ}{\alpha }\overset{ˆ}{\beta }+(\overset{ˆ}{\alpha }+\overset{ˆ}{\beta }+1)(\overset{ˆ}{\alpha }+\overset{ˆ}{\beta }{)}^{2}\frac{{\overset{ˆ}{p}}_{i,RE}\left(1-{\overset{ˆ}{p}}_{i,RE}\right)}{n_{i}}}$	Sample means of binary outcomes
Beta-Binomial-Jeffreys^a^	$\frac{\overset{ˆ}{\alpha }\overset{ˆ}{\beta }}{\overset{ˆ}{\alpha }\overset{ˆ}{\beta }+(\overset{ˆ}{\alpha }+\overset{ˆ}{\beta }+1)(\overset{ˆ}{\alpha }+\overset{ˆ}{\beta }{)}^{2}\frac{{\overset{ˆ}{p}}_{i}^{*}\left(1-{\overset{ˆ}{p}}_{i}^{*}\right)}{n_{i}}}$	Sample means of binary outcomes
Resampling IUR	$\frac{{\overset{ˆ}{\sigma }}_{\text{RIUR}}^{2}-{\overset{ˆ}{\sigma }}_{w,\text{RIUR}}^{2}}{{\overset{ˆ}{\sigma }}_{\text{RIUR}}^{2}}$	Any

*Note*: ${\overset{ˆ}{p}}_{i,FE},{\overset{ˆ}{p}}_{i,RE}$, and ${\overset{ˆ}{p}}_{i}^{*}$ are provider-specific estimates of performance from a fixed effects model, random effects model, and from a Bayesian model with Jeffrey’s prior, respectively. $\overset{‾}{p}$ is the overall sample proportion of patients meeting the measure criteria.

a These methods yield provider-specific estimates.

b These methods use the model-estimated fixed effects for the marginal probability estimate in the delta method approximation.

c These methods use the overall sample proportion as the marginal probability estimate in the delta method approximation.

**TABLE 3:** Sample size summary statistics.

Method	AMM 1	AMM 2	HRF1	HRF2	HRF3	HRF4	HRF5
Providers	129	129	141	141	141	141	141
Smallest sample size	159	159	2	2	1	1	2
Largest sample size	3279	3279	626	589	540	471	1585
Median sample size	651	651	116	111	98	87	183
Sample size IQR	(427, 1078)	(427, 1078)	(71, 182)	(64, 177)	(58, 164)	(53, 143)	(121, 323)

**TABLE 4:** Reliability estimates from different methods.

Method	AMM 1	AMM 2	HRF1	HRF2	HRF3	HRF4	HRF5
Permutation split-sample	0.954	0.932	0.787	0.731	0.668	0.651	0.972
ANOVA^a^	0.960	0.941	0.797	0.743	0.624	0.640	0.958
Hier. Linear^a^	0.961	0.942	0.793	0.754	0.649	0.656	0.966
Hier. Logit 1^a,b^	0.963	0.942	0.789	0.723	0.660	0.699	0.914
Hier. Logit-FE 1^a,b^	0.967	0.943	0.815	0.742	0.702	0.712	0.927
Hier. Logit-RE 1^a,b^	0.967	0.942	0.794	0.705	0.657	0.693	0.917
Hier. Logit 2^a,c^	0.964	0.942	0.827	0.749	0.681	0.714	0.941
Hier. Logit-FE 2^a,c^	0.969	0.943	0.878	0.789	0.740	0.741	0.967
Hier. Logit-RE 2^a,c^	0.969	0.943	0.862	0.756	0.698	0.723	0.962
Beta-Binomial^a^	0.962	0.941	0.813	0.748	0.678	0.704	0.943
Beta-Binomial-FE^a^	0.965	0.940	0.857	0.781	0.725	0.721	0.968
Beta-Binomial-RE^a^	0.965	0.940	0.839	0.748	0.681	0.702	0.964
Beta-Binomial-Jeffreys^a^	0.965	0.940	0.837	0.748	0.708	0.715	0.963
Resampling IUR	0.969	0.954	0.846	0.793	0.705	0.714	0.968

a These methods yield provider-specific estimates; this table shows the median provider-specific reliability estimate.

b These methods use the model-estimated fixed effects for the marginal probability estimate in the delta method approximation.

c These methods use the overall sample proportion as the marginal probability estimate in the delta method approximation.

**FIGURE A1:**
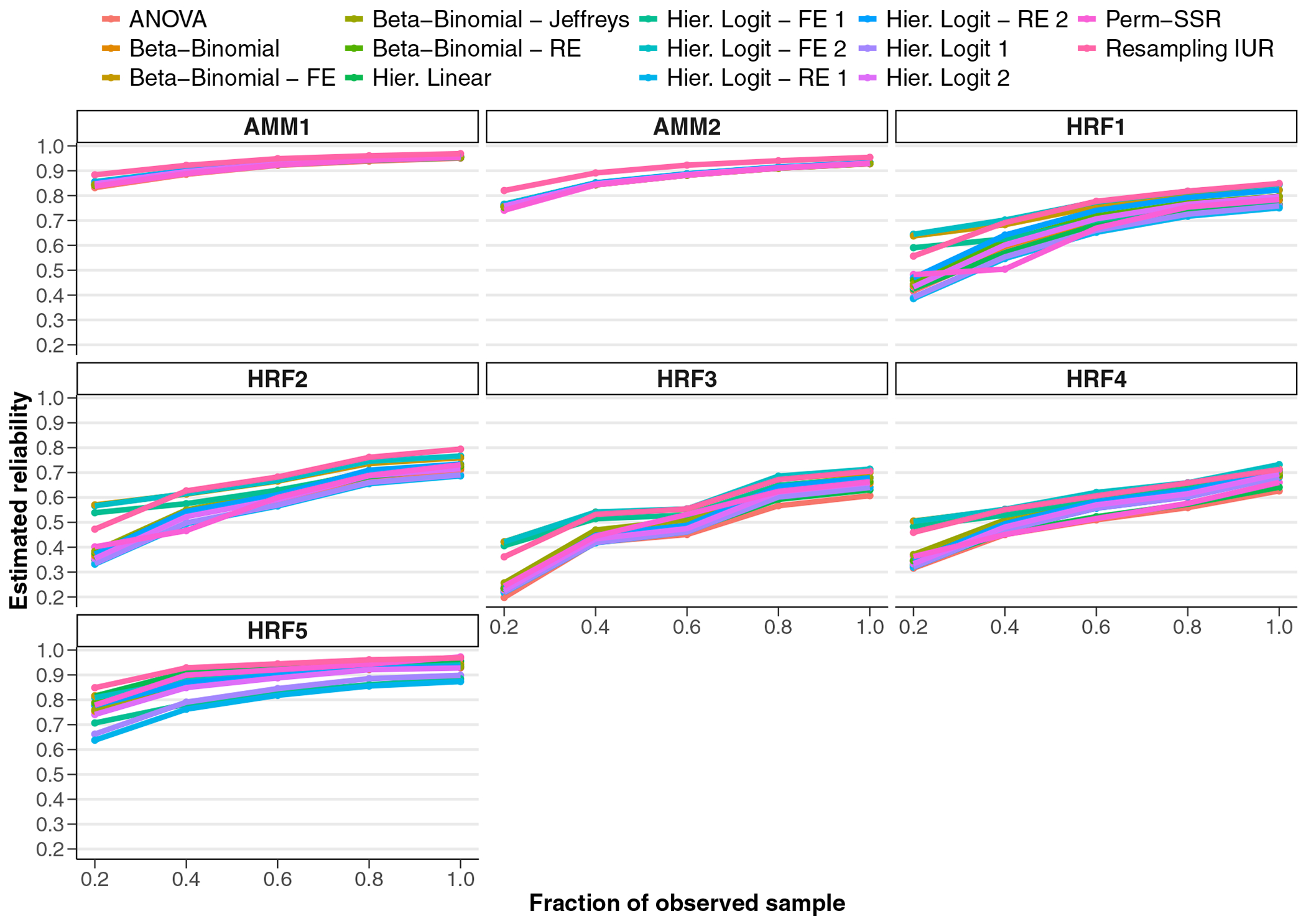
Plots of reliability estimates for each method and for varying sample sizes, corresponding to 20%, 40%, 60%, 80%, and 100% of the observed data for each medical center.

## Data Availability

The datasets analyzed during the current study were derived from the VA Corporate Data Warehouse and are not publicly available due to identifying nature of patients and providers. However, how data were collected and managed can be shared via the corresponding author on reasonable request.

## APPENDIX A

### A.1 ANOVA

We assume the model

$$Y_{\mathrm{ij}}=Z_{i}+\epsilon_{\mathrm{ij}},$$

where *Z_i_* and *ij* are independent for all *i, j*, *Z*_1_,…, *Z_m_* are independent, and *ε_ij_*, *ε_ik_* are independent for all j≠k. $\mathrm{var}Z_{i}=\sigma_{b}^{2}$ and var $\epsilon_{\mathrm{ij}}=\sigma_{w}^{2}$. We define the following terms, $N=\sum_{i=1}^{m}n_{i};{\overset{‾}{Y}}_{i}=\frac{1}{n_{i}}\sum_{j=1}^{n_{i}}Y_{\mathrm{ij}};\bar{\overset{‾}{Y}}=\frac{1}{N}\sum_{i=1}^{m}\sum_{j=1}^{n_{i}}Y_{\mathrm{ij}};\mathrm{MSB}=\frac{1}{m-1}\sum_{i=1}^{m}n_{i}{\left({\overset{‾}{Y}}_{i}-\bar{\overset{‾}{Y}}\right)}^{2};$ and $\mathrm{MSW}=\frac{1}{N-m}\sum_{i=1}^{m}\sum_{j=1}^{n_{i}}{\left(Y_{\mathrm{ij}}-\bar{Y_{i}}\right)}^{2}$.We note that

$$\text{var}\left({\bar{Y}}_{i}\right)=\sigma_{b}^{2}+\sigma_{w}^{2}/n_{i}\;\text{var}(\bar{\bar{Y}})=\frac{\sigma_{b}^{2}}{N^{2}}\overset{m}{\underset{i=1}{\sum }}n_{i}^{2}+\frac{\sigma_{w}^{2}}{N}\;\text{cov}\left(Y_{\mathrm{ij}},{\bar{Y}}_{i}\right)=\text{cov}\left(Z_{i}+\epsilon_{\mathrm{ij}},Z_{i}+\frac{1}{n_{i}}\overset{n_{i}}{\underset{k=1}{\sum }}\epsilon_{ik}\right)\;=\sigma_{b}^{2}+\sigma_{w}^{2}/n_{i}\;\text{cov}\left({\bar{Y}}_{k},\bar{\bar{Y}}\right)=\text{cov}\left(Z_{k}+\frac{1}{n_{k}}\overset{n_{k}}{\underset{j=1}{\sum }}\epsilon_{kj},\frac{1}{N}\overset{m}{\underset{i=1}{\sum }}n_{i}Z_{i}+\frac{1}{N}\overset{m}{\underset{i=1}{\sum }}\overset{n_{i}}{\underset{j=1}{\sum }}\epsilon_{\mathrm{ij}}\right)\;=\frac{1}{N}\left(n_{k}\sigma_{b}^{2}+\sigma_{w}^{2}\right)\;\text{var}\left({\bar{Y}}_{i}-\bar{\bar{Y}}\right)=\sigma_{b}^{2}\left(1+\frac{1}{N^{2}}\overset{m}{\underset{i=1}{\sum }}n_{i}^{2}-\frac{2n_{i}}{N}\right)+\sigma_{w}^{2}\left(\frac{1}{n_{i}}-\frac{1}{N}\right)\;\text{var}\left(Y_{\mathrm{ij}}-{\bar{Y}}_{i}\right)=\frac{n_{i}-1}{n_{i}}\sigma_{w}^{2}.$$

Based on this, we have

$$E[\text{MSB}]=\frac{1}{m-1}\overset{m}{\underset{i=1}{\sum }}n_{i}E\left[{\left(\bar{Y_{i}}-\bar{\bar{Y}}\right)}^{2}\right]\;=\frac{1}{m-1}\overset{m}{\underset{i=1}{\sum }}n_{i}\text{var}\left(\bar{Y_{i}}-\bar{\bar{Y}}\right)\;=n_{0}\sigma_{b}^{2}+\sigma_{w}^{2}$$

where $n_{0}=(m-1{)}^{-1}\left(N-\sum_{i=1}^{m}n_{i}^{2}/N\right)$, and

$$E[\text{MSW}]=\frac{1}{N-m}\overset{m}{\underset{i=1}{\sum }}\overset{n_{i}}{\underset{j=1}{\sum }}E\left[{\left(Y_{\mathrm{ij}}-\bar{Y_{i}}\right)}^{2}\right]\;=\frac{1}{N-m}\overset{m}{\underset{i=1}{\sum }}\overset{n_{i}}{\underset{j=1}{\sum }}\text{var}\left[Y_{\mathrm{ij}}-\bar{Y_{i}}\right]\;=\sigma_{w}^{2}.$$

Solving for $\sigma_{b}^{2}$ and $\sigma_{w}^{2}$, we have

$$\sigma_{b}^{2}=\frac{E[\mathrm{MSB}]-E[\mathrm{MSW}]}{n_{0}}\;\sigma_{w}^{2}=E[\mathrm{MSW}].$$

### A.2 Delta method approximations

In the hierarchical logistic model, we have $\mathrm{var}\left(E\left[{\overset{‾}{Y}}_{i}|Z_{i}\right]\right)=\mathrm{var}\left({\mathrm{logit}}^{-1}\left(Z_{i}\right)\right)$. We are given that $\sqrt{n_{i}}\left(Z_{i}-\mu \right)\overset{D}{\to }N\left(0,n_{i}\sigma_{b,\mathrm{HLM}}^{2}\right).$ Let $g(t)={\mathrm{logit}}^{-1}(t).$ Then $g^{\prime }(t)={logit}^{-1}(t)\left(1-{logit}^{-1}(t)\right).$ So, $\sqrt{n_{i}}\left(g\left(Z_{i}\right)-g(\mu )\right)\overset{D}{\to }N\left(0,n_{i}\sigma_{b,\mathrm{HLM}}^{2}{\left(g^{\prime }(\mu )\right)}^{2}\right.$. This implies $\mathrm{var}\left(E\left[{\overset{‾}{Y}}_{i}|Z_{i}\right]\right)\approx \sigma_{b,\mathrm{HLM}}^{2}{\left({logit}^{-1}(\mu )\left(1-{logit}^{-1}(\mu )\right)\right)}^{2}$.

We also have $\mathrm{var}\left({\overset{‾}{Y}}_{i}|Z_{i}\right)=n_{i}^{-1}{logit}^{-1}\left(Z_{i}\right)\left(1-{logit}^{-1}\left(Z_{i}\right)\right)$. Let $h(t)={logit}^{-1}(t)\left(1-{logit}^{-1}(t)\right)$. Using the delta method approximation, $E\left[\mathrm{var}\left({\overset{‾}{Y}}_{i}|Z_{i}\right)\right]\approx n_{i}^{-1}{logit}^{-1}(\mu )\left(1-{logit}^{-1}(\mu )\right)$.

### A.3 Beta-binomial derivations

In the Beta-Binomial model, for the between-provider variance calculation, we have that $E\left[n_{i}{\overset{‾}{Y}}_{i}|Z_{i}\right]=E\left[n_{i}Z_{i}|Z_{i}\right]=n_{i}Z_{i}$. So, $E\left[{\overset{‾}{Y}}_{i}|Z_{i}\right]=Z_{i}$. This implies that $\mathrm{var}\left(E\left[{\overset{‾}{Y}}_{i}|Z_{i}\right]\right)=\mathrm{var}\left(Z_{i}\right)=\alpha \beta /(\alpha +\beta {)}^{2}(\alpha +\beta +1)$.

For the within-provider variance calculation, we have that $\mathrm{var}\left(n_{i}{\overset{‾}{Y}}_{i}|Z_{i}\right)=n_{i}Z_{i}\left(1-Z_{i}\right)$. So, $\mathrm{var}\left({\overset{‾}{Y}}_{i}|Z_{i}\right)=n_{i}^{-1}Z_{i}\left(1-Z_{i}\right)$. This implies that $E\left[\mathrm{var}\left({\overset{‾}{Y}}_{i}|Z_{i}\right)\right]=n_{i}^{-1}\left(E\left[Z_{i}\right]-E\left[Z_{i}^{2}\right]\right)=\alpha \beta /n_{i}(\alpha +\beta )(\alpha +\beta +1)$.

Note that $\mathrm{var}\left({\overset{‾}{Y}}_{i}\right)=\mathrm{var}\left(E\left[{\overset{‾}{Y}}_{i}|Z_{i}\right]\right)+E\left[\mathrm{var}\left({\overset{‾}{Y}}_{i}|Z_{i}\right)\right]=\alpha \beta \left(\alpha +\beta +n_{i}\right)/n_{i}(\alpha +\beta {)}^{2}(\alpha +\beta +1)$.

### A.4 Beta-binomial sample code

The joint log-likelihood for the Beta-Binomial model is given by:

log⁡Lα,β;x1,…,xm=∑i=1mlog⁡Γ(α+β)-log⁡Γ(α)-log⁡Γ(β)+log⁡nixi+log⁡Γα+xi+log⁡Γβ+ni-xi-log⁡Γα+β+ni,

where *n_i_* is the number of observations for provider *i*, *x_i_* is the number of observations meeting the measure criteria (ie, $x_{i}=n_{i}{\overset{‾}{y}}_{i}$), and Γ(·) is the gamma function.

calcBetaBin <- function(n, x){

  # function to calculate negative log-likelihood

  neg.loglik <- function(v){

    -sum(lgamma(v[1] + v[2]) − lgamma(v[1]) − lgamma(v[2]) +

    lchoose(n, x) + lgamma(v[1] + x) + lgamma(v[2] + n − x) −

   lgamma(v[1] + v[2] + n))

  }

  # calculate method of moments estimates for starting values

  m1 = mean(x)

  m2 = mean(x ^2)

  median.n = median(n)

  a.mom = (median.n*m1 - m2) / (median.n *(m2/m1 - m1 - 1) + m1)

  b.mom = ((median.n - m1) * (median.n - m2/m1)) / (median.n*(m2/m1 - m1 - 1) + m1)

  a0 = max(a.mom, .5)

  b0 = max(b.mom, .5)

  # maximize negative log-likelihood

  theta = optim(c(a0, b0), neg.loglik,

        method = ‘L-BFGS-B’, lower = c(1e-6,1e-6))$par

  a = theta[1]

  b = theta[2]

  # calculate variance ratio

  var.b = a * b/ (a + b + 1) / (a + b) ^ {2}

  est = n/ (a + b + n)

  results <- list(est.reliability = est,

            var.b = var.b,

            alpha = a,

            beta = b)

  return(results)

}

### A.5 Resampling IUR sample code

calcResamplingIUR <- function(df, resamples){

  providers = unique(df$provider)

  n.providers = length(providers)

  n.pts <- aggregate(id ~ provider, data = df, length)[,2]

  n0 = 1 / (n.providers - 1) * (sum(n.pts) - sum(n.pts ^ 2) / sum(n.pts))

  gm = mean(df$y)

  provider.means0 = aggregate(y ~ provider, data = df, mean)$y

  var.total = 1/(n0*(n.providers - 1)) * sum(n.pts * (provider.means0 - gm) ^ 2)

  provider.means = matrix(data = NA, nrow = n.providers, ncol = resamples)

  for (j in 1:resamples){

   # take a bootstrap resample within each provider separately

   df.resample = data.frame()

   for (i in 1:n.providers){

    provider.df <- df[df$provider == providers[i], ]

    provider.df.resample <-

      provider.df[sample(nrow(provider.df), nrow(provider.df), replace = T), ]

    df.resample <- rbind(df.resample, provider.df.resample)

   }

   # calculate measure by provider

   provider.means[,j] = aggregate(y ~ provider, data = df.resample, mean)$y

  }

  bootstrap.means = apply(provider.means, 1, mean)

  bootstrap.sqrd.resid = apply(provider.means, 2, function(x) (x - bootstrap.means)

  bootstrap.var = 1/(resamples - 1) * apply(bootstrap.sqrd.resid, 1, sum)

  var.w = sum((n.pts - 1) * bootstrap.var) / (sum(n.pts) - n.providers)

  IUR = (var.total - var.w) / var.total

  results = list(var.b = var.total - var.w,

           var.w = var.w,

           var.total = var.total,

           IUR = IUR)

  return(results)

}

### A.6 Sensitivity analyses

Many methods of estimating reliability yield provider-specific estimates of reliability rather than a single overall estimate. For our main analyses, we chose the median provider-specific reliability estimate to compare with other methods. As a sensitivity analysis, we repeated our subsampling analyses using the arithmetic mean provider-specific reliability estimate. Findings were similar as shown in Figure A1.

## Abbreviations:

AMM: antidepressant medication management
ANOVA: analysis of variance
CMS: center for medicare and medicaid services
HRF: high risk flag
IUR: interunit reliability
NQF: national quality forum
PHQ-9: patient health questionnaire-9
VA: veterans health administration
